# CSNOMA: Carrier Sense Non-Orthogonal Multiple Access

**DOI:** 10.3390/s20185024

**Published:** 2020-09-04

**Authors:** Dong Min Kim, Seong-Lyun Kim

**Affiliations:** 1 Department of Internet of Things, SCH Media Labs, Soonchunhyang University, Asan 31538, Korea; 2 School of Electrical and Electronic Engineering, Yonsei University, Seoul 03722, Korea; slkim@yonsei.ac.kr

**Keywords:** random access, interference cancellation, distributed MAC, area spectral efficiency

## Abstract

In this paper, we investigate the possibility of the cross-layer design of a distributed random access scheme with considering physical (PHY) and multiple access control (MAC) layers, which utilizes the interference cancellation technique. In this regard, we propose a new multiple access protocol, named carrier sense non-orthogonal multiple access (CSNOMA). We consider the spatially randomly distributed interferers to realistically capture the effect of interference. The proposed protocol shows better area spectral efficiency than carrier sense multiple access (CSMA), as the node density increases. We also present a practical signaling design compatible with IEEE 802.11 DCF mode.

## 1. Introduction

With growing demands for wireless connectivity, a multiple access control (MAC) scheme supporting a large number of networked devices becomes increasingly crucial. Cisco predicts that the number of wireless networked devices will be 13.1 billion in 2023 [1]. In line with this trend, it is expected that new kinds of networks will arise. For example, Tactile Internet requires ultra-low delays and extremely high availability and reliability to enable transmission of tactile information [2]. The stringent requirements of Tactile Internet will drive to innovation of MAC design. The radio resources can be used by centralized (scheduling) or distributed MAC (random access). Because of its complexity, the latter is more suitable for an ultra-dense environment. There are two representative random access schemes: ALOHA and carrier sense multiple access (CSMA). To measure the performance of MAC with emphasis on the number of supporting users, an area spectral efficiency (ASE) is widely adopted [3]. ASE is the product of the successfully transmitting user density and the data rate per node. In the ALOHA system, to maximize ASE, the transmission probability is optimized [4]. On the other hand, the carrier sensing threshold is optimized in the CSMA system [5]. A multiuser interference is an avoiding factor for both schemes.

“When a station is hidden from either the transmitting or the receiving station, by detecting just one frame among the RTS and CTS frames, it can suitably delay further transmission, and thus avoid collision.” The quote is in the seminal work by Bianchi [6]. For the multiple access control (MAC) protocol, designers and researchers have widely accepted the idea of avoiding interference.

Breaking away from the notion of a traditional thinking, we propose a new random access protocol, Carrier Sense Non-Orthogonal Multiple Access (CSNOMA). It is inspired by Non-Orthogonal Multiple Access (NOMA) [7], which has recently been in the spotlight in cellular communication [8,9]. We sketch out the operation of CSNOMA briefly. Let us assume that a transmitter/receiver pair wants to communicate with each other in the wireless network. The transmitter sends a signal before the data transmission to reserve a radio channel. If the signal is correctly received, the corresponding receiver sends a response to the transmitter. We apply the hierarchical modulation on the response. If a node, among the neighbor nodes who overhear the signal handshaking of the communication pair, can decode the hierarchical modulation, additional information can be obtained. This node decides whether or not to transmit based on the additional information. Otherwise, the node should shutdown if the node just decodes the original response. In this way, the receiver gives a transmission chance to the neighbor node who shows the best channel gain. After this preprocessing, the transmitter and the selected neighbor node transmit their data simultaneously. The receiver carries out interference cancellation and detection. If the transmission succeeds, the receiver sends ACK to the transmitter.

The wireless sensor network (WSN) is a representative field where distributed MAC is used. Because WSN uses devices with small transmission power, the transmission distance is often short. In order to increase the distance of information transmission, many routing protocols have been studied, and the MAC researchers have mainly focused on the combination of the MAC and the network layer, and thus more energy saving techniques have been studied [10,11]. The distributed MAC protocol can be designed under the idea of utilizing interference to improve ASE. There are reception/transmission techniques utilizing interference in the physical (PHY) layer, such as interference cancellation (IC) [12], interference alignment (IA) [13], dirty paper coding (DPC) [14], analog network coding (ANC) [15], physical network coding (PNC) [16], full-duplex radio [17,18,19], etc. There is a lack of research on the MAC design supporting these advanced PHY layer techniques. There are a few studies that apply the technology used in cellular communication to a distributed MAC, including the works in [20,21]. The authors of [20] utilize CDMA-like technology, a technology used in the 2nd generation cellular communication, in MAC design for mobile ad hoc network. The authors of [21] investigate the possibility of OFDMA, a technology used in the 3rd generation cellular communication, in MAC protocol for cyber-physical systems. We investigate the possibility of cross-layer design of distributed random access scheme with PHY and MAC layers. The main contributions of this paper are summarized as follows.

- We propose a new multiple access protocol, carrier sense non-orthogonal multiple access (CSNOMA), which utilizes the interference cancellation technique in the sense of cross-layer design with considering physical (PHY) and multiple access control (MAC) layers.
- We show the area spectral efficiency performance of the proposed scheme is better than CSMA/CA, as the node density increases.
- We present a practical signaling design which is compatible with IEEE 802.11 DCF mode.

The rest of the paper is organized as follows. Section 2 presents previous studies related to this research. We describe the system model in Section 3. We propose and analyze CSNOMA in Section 4. In Section 5, we verify the performance of the proposed scheme with numerical results. Section 6 concludes the paper.

## 2. Related Work

The characteristics of scheduling schemes utilizing IC are investigated in [22,23,24,25,26]. However, they consider the low dense network, so the performance gain is not properly investigated as increasing the nodes in the network. The authors of [27] investigate the network scaling law when multipacket reception is adopted. However, they simplify the interference cancellation condition. The authors of [28,29,30] consider the improvement in transmission capacity obtainable with IC. They assume that all interfering signals close to the receiver can be canceled even if the signal strength is under the target SINR threshold. They did not consider the MAC design issue either.

In [31], the authors are motivated by the idea of cross-layer design of wireless networks. The authors of [31] examine one aspect of the interaction between the physical and medium access control layers, in particular, the impact of signal processing techniques that enable multipacket reception on the throughput and design of random access protocols. The authors of [32,33,34] also consider the multiple access control design with multipacket reception.

IC is a practical interference management technique, which is actively discussed as a part of enhanced Inter-Cell Interference Coordination (eICIC) for LTE Advanced (LTE-A) heterogeneous networks [35] and NOMA [8]. IC could also be utilized in distributed random access schemes because IC is a promising technique when the received signal strengths from the different interferers vary severely and it happens frequently in distributed wireless networks [36,37,38]. In the ALOHA system, the concurrent transmitting nodes are randomly distributed in the space, and interferers could exist closer than the desired transmitter. This interferer causes the outage, i.e., signal-to-interference-plus-noise-ratio (SINR) at the receiver is below the target SINR threshold. However, if the receiver has IC capability, the above situation would even be favorable because the interfering signal is easier to cancel as the interferer is getting closer to the receiver. In the CSMA system, the above situation could happen if the sensing range is short, namely, the hidden node problem. Using IC, the hidden node problem can be resolved, and higher ASE could be achieved than that of the conventional CSMA system. In [39], the authors noted that current CSMA design is not suitable to combining IC capability. It also motivates us to design CSNOMA in an ultra-dense network to see if CSNOMA can enhance ASE and other performance metrics. To maximize ASE, we combine the random access schemes with IC capability. This work quantifies the performance improvement on distributed MAC by using IC and provides the insight to design a new random access scheme. Table 1 summaries the aim, proposed solution, pros, cons of the related work.

## 3. System Model

The key mathematical notations used in this paper are listed in Table 2.

### 3.1. Network Model

**Figure 1:**
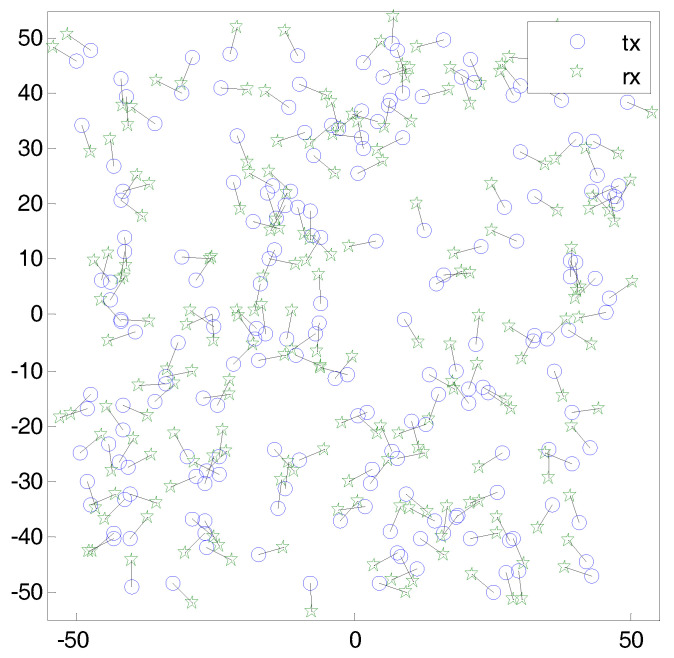
A snapshot of wireless ad hoc network, where transmitters are randomly located according to a 2-dimensional homogeneous Poisson point process (PPP) with intensity λ. Each transmitter *i* has a dedicated receiver k(i), who is situated at a distance $r_{t}$ from the transmitter with random direction.

Assume that a wireless network where transmitters are randomly located according to a 2-dimensional homogeneous Poisson point process (PPP) with intensity λ. All distance units are in meters. Each transmitter *i* has a dedicated receiver k(i) that is situated at a distance $r_{t}$ from the transmitter with random direction. Figure 1 shows a snapshot of the wireless network. According to the displacement theorem [40], receivers also follow a homogeneous PPP. All transmitters have infinite backlogged data to transmit. A common channel is shared by the entire network. Thus, all concurrent transmissions act as interference. All transmitters emit the packet with constant transmission power *P*. The received signal power at a node *j* from a node *i* is denoted by $g_{i,j}d_{i,j}^{-\alpha }P$, where $g_{i,j}$ is an exponential random variable with unit mean and reflects the effect of Rayleigh fading. The value $d_{i,j}$ denotes the distance between nodes *i* and *j* with the path loss exponent α. We assume that the time is slotted and synchronized so that transmissions start with the beginning of time slot and continue during the slot length. The transmitter/receiver pair can vary over the time, but it is assumed that the topology and channel gains are fixed during a slot duration. We assume that the network is interference-limited and the noise at receiver can be ignored. An instantaneous signal-to-interference-ratio (SIR) of a typical receiver, $\gamma_{k(i)}$, which is a measure of the impact of the interference, is given by

(1) $$\gamma_{k\left(i\right)}=\frac{g_{i,k\left(i\right)}r_{t}^{-\alpha }P}{\sum_{u\in T_{i}}g_{u,k\left(i\right)}d_{u,k\left(i\right)}^{-\alpha }P},$$

where $T_{i}$ denotes the set of simultaneously transmitting nodes with the node *i*. The MAC scheme confirms the set $T_{i}$. For the ALOHA-like random access scheme, the spatial pattern can be successfully modeled by PPP [4]. That is, $T_{i}$ is an independently thinned point process of the original node set.

For a given target SIR β, the transmission is successful if the SIR at the receiver k(i) is greater than or equal to β. The instantaneous data rate of the transmitter *i* is a function of the target SIR. We use the Shannon’s formula $\log_{2}\left(1\;+\;\beta \right)$ with a unit bandwidth. Assume that all receivers are able to fulfill the successive interference cancellation (SIC). In next subsection, we will describe the interference cancellation model.

### 3.2. Interference Cancellation Model

In [41], it is shown that canceling just the strongest interferer shows huge performance improvement. The aggregate interference from the randomly located concurrent transmitters reduces the SIR of the desired signal at the receiver, and can cause the transmission failure. By applying SIC [12], the perished signal can be resuscitated. Among the interfering signals, the strongest interference would be decoded by the receiver if the SIR of the strongest interference is greater than a target threshold, and strong interference signal is extracted from the aggregate interference. Using the remaining signals, the receiver recalculates the SIR of the desired signal, and if the SIR of the desired signal steps over the target SIR β, the signal can be finally decoded.

**Figure 2:**
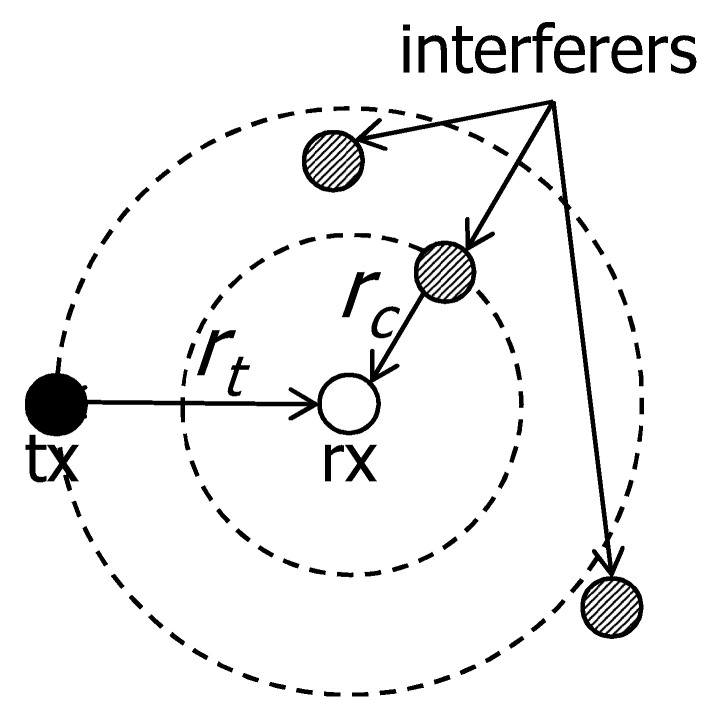
Example of cancelable scenario. There are one communication pair with communication distance $r_{t}$ and the target SIR is β. There are three interferers. The interfering signal within the distance $r_{c}=\beta^{-\frac{1}{\alpha }}r_{t}$ would be cancelable by the rx. On the other hand, the other two interfering signals cannot be canceled.

Figure 2 illustrates an example of the cancelable interference. There are one communication pair with communication distance $r_{t}$ and the target SIR is β. There are three interferers. We define that a cancelable distance $r_{c}$ is the maximum distance that the transmission of the interferer within $r_{c}$ can be canceled by the receiver. If the only one interferer is at a distance *r* from the receiver, in order to decode the signal from the interferer, it should satisfy following condition.

(2) $$\begin{array}{c} \frac{r^{-\alpha }P}{r_{t}^{-\alpha }P}\geq \beta ,\\ r\leq \beta^{-\frac{1}{\alpha }}r_{t}. \end{array}$$

Thus, the interfering signal within the distance $r_{c}=\beta^{-\frac{1}{\alpha }}r_{t}$ would be cancelable by the rx. On the other hand, the other two interfering signals cannot be canceled. If the target SIR increases, the cancelable region shrinks.

In the fading environments, due to the randomness of the channel gain, not all interference within $r_{c}$ can be canceled, and not all interference farther than $r_{c}$ cannot be canceled. We denote the values $g_{c}$ and $g_{t}$ as the channel gains of channel from the interferer at a distance *r* and the desired transmitter at a distance $r_{t}$, respectively. To cancel the interference signal traveled distance *r*, the following condition should be satisfied,

(3) $$\frac{g_{c}r^{-\alpha }P}{g_{t}r_{t}^{-\alpha }P+I^{\prime }}\geq \beta ,$$

where $I^{\prime }$ denotes the aggregate received power excluding the desired signal and the interfering signal from a distance of *r*. In the next section, we explain a new random access scheme using IC and quantify the performance improvement.

## 4. Carrier Sense Non-Orthogonal Multiple Access (CSNOMA)

### 4.1. Proposed Protocol

**Figure 3:**
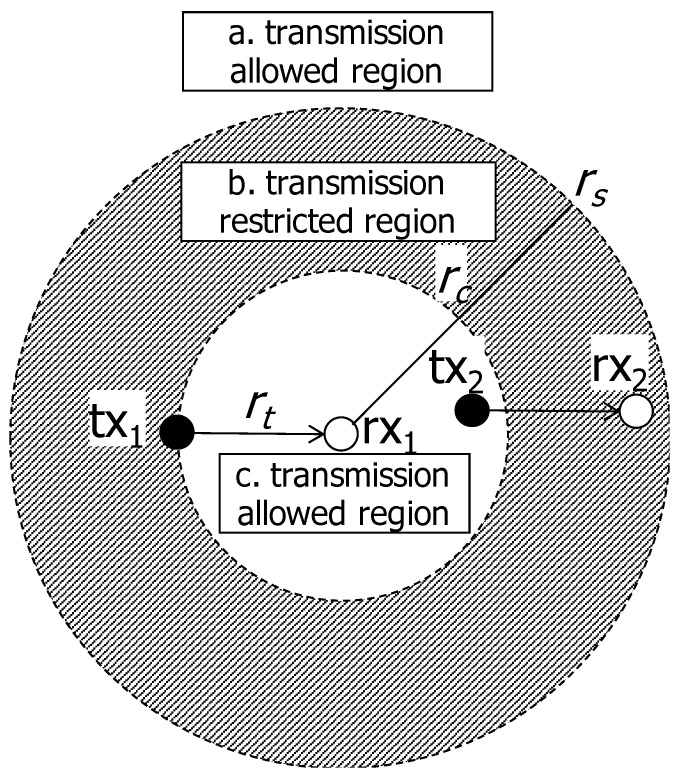
Transmission allowed/restricted region. When the ${\mathrm{tx}}_{1}$ has a chance to transmit, the another transmitter (${\mathrm{tx}}_{2}$) within the circular region of radius $r_{c}$ (region c, inside of the inner circle) is also allowed to transmit. The transmitters in the annulus region between $r_{c}$ and $r_{s}$ (region b, shaded region in the figure) are still restricted to transmit, and the transmitters farther than $r_{s}$ (region a, outside of the outer circle) can transmit if the channel is sensed as idle.

Under the CSMA regime, a node in the network can transmit its data when the channel is sensed as idle. The conventional CSMA-like random access scheme prohibits the transmitters in the proximity from transmitting concurrently to increase the transmission success probability. As the node density increases, more nodes struggle to seize the transmission opportunity due to the conservative behavior of CSMA. To resolve this situation, we take a radical approach; even though the channel is sensed as busy, the transmission is allowed and incurred interference is canceled by SIC. Figure 3 shows the allowed/restricted region. When the ${\mathrm{tx}}_{1}$ has a chance to transmit, the another transmitter (${\mathrm{tx}}_{2}$) within the circular region of cancelable distance $r_{c}$ (region c, inside of the inner circle) is also allowed to transmit. The transmitters in the annulus region between $r_{c}$ and $r_{s}$ (region b, shaded region in the figure) are restricted to transmit, and the transmitters farther than $r_{s}$ (region a, outside of the outer circle) can transmit if the channel is sensed as idle. Classification of allowed/restricted transmission areas according to the distance from the receiver is depicted in Table 3.

We explain the operation of CSNOMA. Let us assume that a typical transmitter/receiver pair wants to communicate with each other in the wireless network of a number of transmitter/receiver pairs. If the wireless channel is sensed as idle during the specific time, such as DIFS in IEEE 802.11 DCF, the typical transmitter goes in the contention period. Then, the typical transmitter takes random backoff and defer its transmission until the backoff counter is expired. When the typical transmitter finally reaches the time to send, the transmitter sends a request-to-send (RTS) signal before the data transmission to reserve a radio channel. If RTS is correctly received, the corresponding receiver sends clear-to-send (CTS) to the transmitter. To enable CSNOMA maintaining the coherent signaling structure of IEEE 802.11, we apply the hierarchical modulation ([42,43]) on the CTS control message. The neighboring nodes can overhear the RTS/CTS handshaking and may obtain hierarchically modulated additional information or only basic information according to each channel state. If the node obtained additional information it can be judged that the channel gain between the typical receiver is relatively fine because the distance from the typical receiver is close. If the node does not obtain additional information, the CTS frame is decoded, as the usual IEEE 802.11 protocol works and the node will sleep according to the Network Allocation Vector (NAV). Thus, the typical receiver provides a transmission opportunity to the adjacent node that can perform IC well. After this preprocessing, the typical transmitter and the selected adjacent node transmit their data at the same time. The typical receiver extracts the signal of the adjacent node from the aggregate interference through IC, and try to decode the signal originally intended to be received with the reduced aggregate interference. The typical receiver informs the typical transmitter of the result. If the transmission is not successful, the contention window size for the retransmission increases exponentially, i.e., binary exponential backoff is applied.

To quantify the network-wide performance of CSNOMA, we analyze ASE of CSNOMA using the stochastic geometry in the next subsection.

### 4.2. Area Spectral Efficiency Analysis

According to the authors of [44], if the carrier sensing range is $r_{s}$, the transmitting node density is $\lambda_{t}$ is

(4) $$\lambda_{t}=\frac{1-\exp\left(-\lambda \pi r_{s}^{2}\right)}{\pi r_{s}^{2}}.$$

The additional transmitting nodes are located within the distance $r_{c}$ of the carrier sensing based transmitting nodes. By using void probability [45], the additional transmitting node density is

(5) $$\lambda_{a}=\lambda_{t}\left(1-\exp\left(-\lambda \pi r_{c}^{2}\right)\right).$$

We define that the probability $p_{s}^{\text{non-IC}}$ is a transmission success probability of a typical transmitter without additional transmission. Henceforth, the path loss exponent α is assumed as 4, which is validated for dense urban area. Then, the success probability $p_{s}^{\text{non-IC}}$ is

(6) $$p_{s}^{\text{non-IC}}=\exp\left(-\pi \left(\lambda_{t}+\lambda_{a}\right)\sqrt{\beta }r_{t}^{2}\arctan\left(\frac{\sqrt{\beta }r_{t}^{2}}{r_{s}^{2}}\right)\right).$$

The derivation of Equation (6) is given in Appendix A.

The probability $p_{s}^{\mathrm{IC}}$ is a transmission success probability of a typical transmitter with an additional transmission within the distance $r_{c}$. Then, a lower bound of the success probability $p_{s}^{\mathrm{IC}}$ is

(7) $$\begin{array}{cc} p_{s}^{\mathrm{IC}} & \geq \int_{0}^{\beta^{-\frac{1}{\alpha }}r_{t}}\exp\left(-\pi \left(\lambda_{t}+\lambda_{a}\right)\sqrt{\beta }r_{t}^{2}\arctan\left(\frac{\sqrt{\beta }r_{t}^{2}}{r_{s}^{2}}\right)\right)\\ \; & \;\times \exp\left(-\pi \left(\lambda_{t}+\lambda_{a}\right)\sqrt{\beta }r_{a}^{2}\arctan\left(\frac{\sqrt{\beta }r_{a}^{2}}{r_{s}^{2}}\right)\right)\frac{2\left(\lambda \right)\pi r_{a}r_{t}^{\alpha }}{\beta r_{a}^{\alpha }+r_{t}^{\alpha }}\\ \; & \;\times \exp\left(-\left(\lambda \right)\pi r_{a}^{2}\right)dr_{a}. \end{array}$$

The derivation of Equation (7) is given in Appendix B.

The probability $p_{s}^{\mathrm{add}}$ is a transmission success probability of the additionally transmitting neighbor node of the typical transmitter. Then, a lower bound of success probability $p_{s}^{\mathrm{add}}$ is

(8) $$\begin{array}{cc} p_{s}^{\mathrm{add}} & \geq \exp\left(-\pi \left(\lambda_{t}+\lambda_{a}\right)\sqrt{\beta }r_{t}^{2}\arctan\left(\sqrt{\beta }\right)\right)\exp\left(-\left(\lambda_{t}+\lambda_{a}\right)\pi r_{t}^{2}\right)\\ \; & +\int_{0}^{\beta^{-\frac{1}{\alpha }}r_{t}}\exp\left(-\pi \left(\lambda_{t}+\lambda_{a}\right)\sqrt{\beta }r_{t}^{2}\arctan\left(\frac{\sqrt{\beta }r_{t}^{2}}{r_{a}^{2}}\right)\right)\\ \; & \;\times \exp\left(-\pi \left(\lambda_{t}+\lambda_{a}\right)\sqrt{\beta }r_{a}^{2}\arctan\left(\sqrt{\beta }\right)\right)\\ \; & \;\times \frac{2\left(\lambda_{t}+\lambda_{a}\right)\pi r_{a}r_{t}^{\alpha }}{\beta r_{a}^{\alpha }+r_{t}^{\alpha }}\exp\left(-\left(\lambda_{t}+\lambda_{a}\right)\pi r_{a}^{2}\right)dr_{a} \end{array}$$

Using Equations (6) and (7), the derivation is straightforward.

Now, we can derive ASE of the proposed scheme (CSNOMA) using Equations (6)–(8). ASE η is

(9) $$\begin{array}{cc} \eta & \leq \log_{2}\left(1+\beta \right)\left\{\lambda_{t}\exp\left(-\lambda_{t}\pi r_{c}^{2}\right)p_{s}^{\text{non-IC}}\right.\\ \; & \;+\lambda_{t}\left(1-\exp\left(-\lambda_{t}\pi r_{c}^{2}\right)\right)p_{s}^{\mathrm{IC}}\\ \; & \;\left.+\lambda_{t}\left(1-\exp\left(-\lambda_{t}\pi r_{c}^{2}\right)\right)p_{s}^{\mathrm{add}}\right\}. \end{array}$$

The reason for this is as follows. If no other nodes are within the region of radius $r_{c}$ of the carrier sensing based transmitting node, the transmission success probability is determined by $p_{s}^{\text{non-IC}}$. This situation happens with probability $\exp\left(-\lambda_{t}\pi r_{c}^{2}\right)$. On the other hand, the probability that the nodes exist within the region of radius $r_{c}$ of the given transmitting node is $\left(1-\exp\left(-\lambda_{t}\pi r_{c}^{2}\right)\right)$. Moreover, the transmission success probabilities of the carrier sensing-based transmitting nodes and additional transmitting nodes are $p_{s}^{\mathrm{IC}}$ and $p_{s}^{\mathrm{add}}$, respectively.

As the carrier sensing range expands, all transmit success probabilities, $p_{s}^{\text{non-IC}}$, $p_{s}^{\mathrm{IC}}$, and $p_{s}^{\mathrm{add}}$, increase. At the same time, however, the transmitting node densities $\lambda_{t}$ and $\lambda_{a}$ are reduced. This means that an optimal operating point exists in regarding of the carrier sensing range. We will discuss about an optimal carrier sensing range in the next subsection.

### 4.3. Optimal Carrier Sensing Range

**Figure 4:**
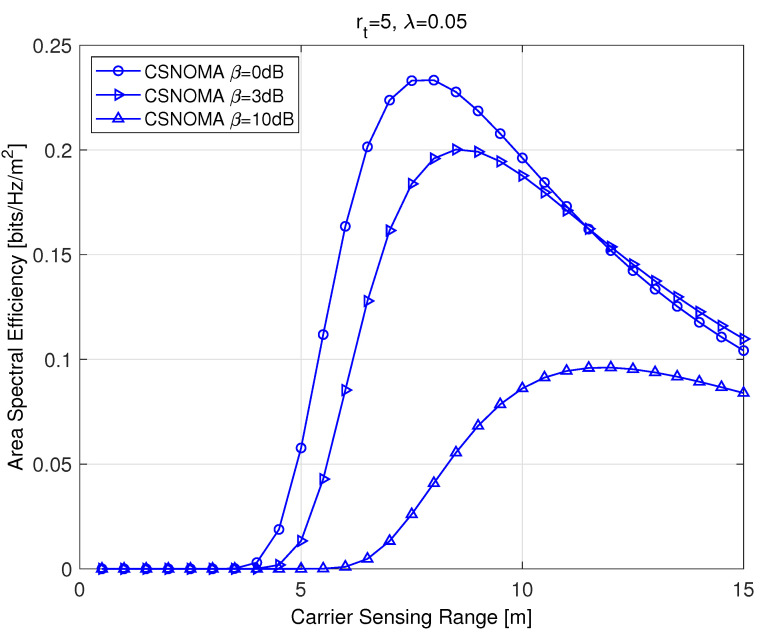
The area spectral efficiency of CSNOMA as a function of the carrier sensing range. The transmission distance $r_{t}$ is 5 and the node density is 0.05. Different target SIR values are used (β = 0 dB and 3 dB).

Figure 4 shows ASE as a function of the carrier sensing range $r_{s}$. The value $r_{s}$ varies from 0 to five times larger than the transmission distance. As shown in Figure 4, an optimal carrier sensing range exists that maximizes ASE. As the target SIR increases, the optimal carrier sensing range becomes larger in order to decrease the aggregate interference level. Equation (9) is a differentiable function of $r_{s}$, and we can apply a simple gradient method to obtain the optimal $r_{s}$ that maximizes ASE:

(10) $$r_{s}^{\left(k+1\right)}=r_{s}^{\left(k\right)}-\omega^{\left(k\right)}▽\eta \left(r_{s}^{\left(k\right)}\right),$$

where $\omega^{\left(k\right)}$ is a step size, where fixed small value (greater than zero and less than 1) or the value found by line search minimization of $\eta (r_{s}-\omega ▽\eta \left(r_{s}\right))$ over ω [46].

### 4.4. Signaling Design

**Figure 5:**
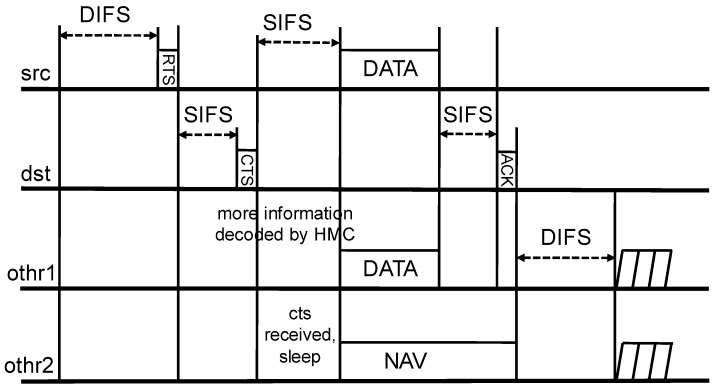
The signaling process of Carrier Sense Non-Orthogonal Multiple Access (CSNOMA).

In this section, we provide the practical signaling process and the control packet frame structure. The proposed signaling scheme is compatible with IEEE 802.11 DCF mode. Figure 5 shows the proposed signaling process of CSNOMA. We try to modify the signaling process of IEEE 802.11 DCF minimally. Thus, it may be possible to colocate the node applying CSNOMA and the node applying IEEE 802.11 DCF.

**Figure 6:**
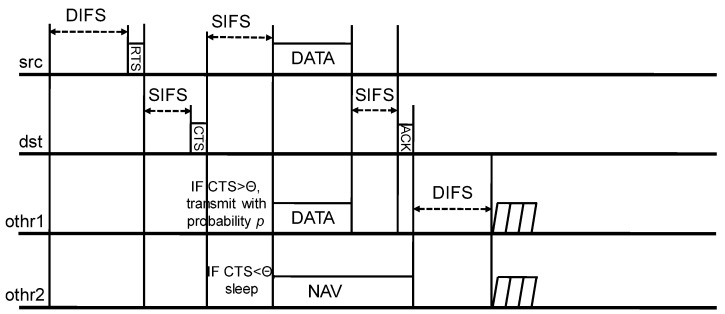
The signaling process for protecting the receiver of additional transmitting nodes.

Figure 6 shows the signaling process for protecting the receiver of additional transmitting nodes as explained in the previous section. The CTS packet that is sent by overheard receiver (rx_othr1) to the silent transmitter (tx_othr1) contains the original form of CTS packet in CSMA. It should be noted that the term CTS means clear-to-send; however, in this case, the term CTS means cease-to-send. That is, the transmitter (tx_othr1) should shutdown the transmission.

#### CTS Frame Format

**Figure 7:**
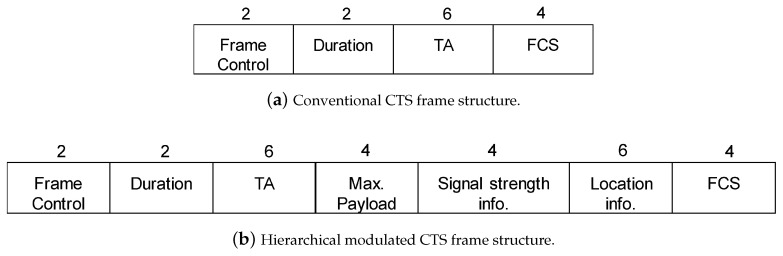
CSNOMA frame structure.

To enable CSNOMA maintaining the same signaling structure of IEEE 802.11, we apply the hierarchical modulation [42] on the CTS control message as shown in Figure 7. If the node that received the CTS frame can decode the hiearchical modulation, additional information can be sent, such as maximum payload size, signal strength related information, and geolocation information, as shown in Figure 7b. This node decides whether to transmit or not based on the additional information. Otherwise, if the node cannot decode the higher modulation part, the remaining operation is the same as the conventional signaling using the frame structure as shown in Figure 7a.

### 4.5. Reactive/Proactive Mode

Although the optimal carrier sensing range was discussed earlier, in reality it is very difficult to always maintain the optimal value. As the communication nodes move the distance between the transmitter and the receiver changes, and the amount of wireless interference received from other nodes also varied. It is also difficult to determine the density of nodes in the network. This is not just a CSNOMA problem. In the CSMA regime, it is not easy to maintain an optimal sensing range. There are problems that arise when the sensing range is not an optimal setting.

**Figure 8:**
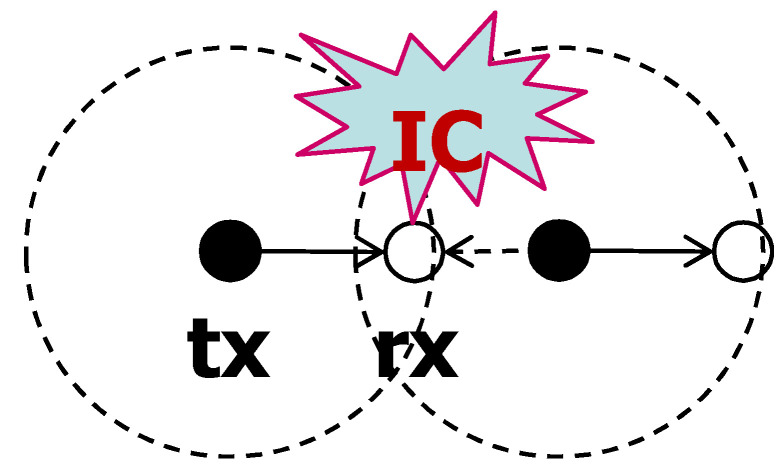
Example for reactive mode operation of CSNOMA. Solution of hidden node problem.

Suppose that the typical receiver and the typical transmitter with a short carrier sensing range are located in the network. Any nodes near the typical receiver, but not in the carrier sensing range of the typical transmitter, can attempt to transmit. Other transmit nodes around the typical receiver interfere with transmission from the typical transmitter. This situation is called the hidden node problem. As explained earlier, CSNOMA deliberately creates this situation. However, it is not necessary if the hidden node problem occurs. If CSNOMA tries to send additional nodes, it aggravates the situation. IC could be applied to resolve the hidden node problem, and not to promote other silent nodes to activate (Figure 8) like in [38]. We define this mode of operation as reactive mode. IC is applied opportunistically and CSNOMA does not make the situation on purpose.

**Figure 9:**
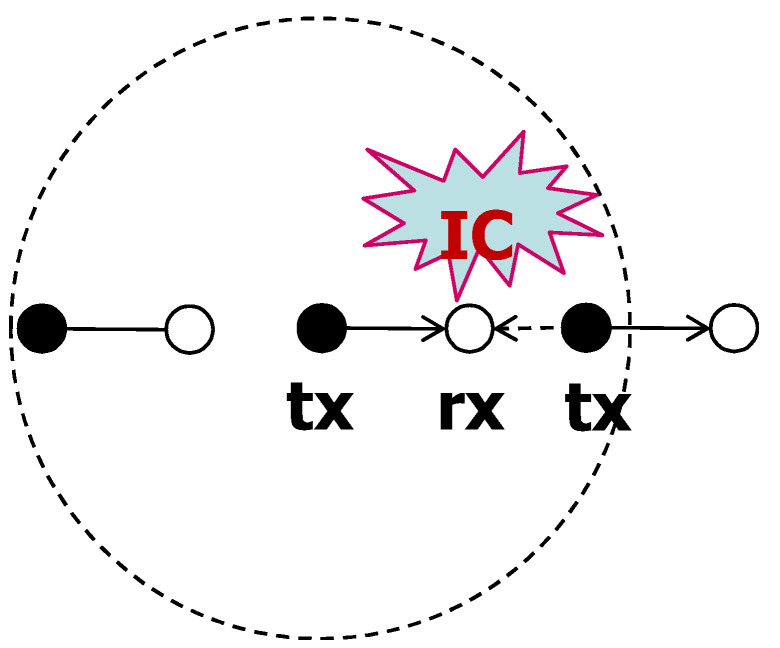
Example for proactive mode operation of CSNOMA. Solution of exposed node problem.

On the other hand, if the carrier sensing range is relatively larger than the communication distance, other transmitting nodes that are far enough away from the typical receiver and do not cause significant interference are also within the sensing range of the typical transmitter and do not transmit. This situation is called the open node problem. In this case, only few nodes have chances to transmit, thus it is desirable to force the silent nodes to wake up and apply interference cancellation (Figure 9). We define this mode as the proactive mode, which is the operation of CSNOMA has been described earlier.

To make CSNOMA operate in reactive mode, disable RTS/CTS handshaking. CSNOMA controls neighboring nodes by performing hierarchical modulation on the CTS frame, so handshaking is not necessary in reactive mode where neighboring nodes do not need to be controlled. As the receiver implementing CSNOMA can still perform IC, it performs IC for hidden node transmission.

As receivers must have IC-related functions, hardware complexity and computation cost increase. Furthermore, as the message decoding process is complicated due to IC, energy consumption increases. However, when the device is running out of energy, it can disable IC-related functions. Then, the device operates in IEEE 802.11 DCF mode, and computation cost and energy consumption can be reduced.

### 4.6. Receiver Protection Mode

One of the problem of CSNOMA is that the additional transmission is not guaranteed to succeed in the reception. To solve this problem, the receiver of the additional transmission behaves more conservatively. If the additional receiver overhears the RTS of conventional transmission node, the additional receiver sends out the CTS packet to its transmitter. If the additional transmitter receives this packet, it should shutdown to avoid collision with the noticed convention transmission.

## 5. Numerical Results

In this section, the performance of CSNOMA is evaluated.

**Figure 10:**
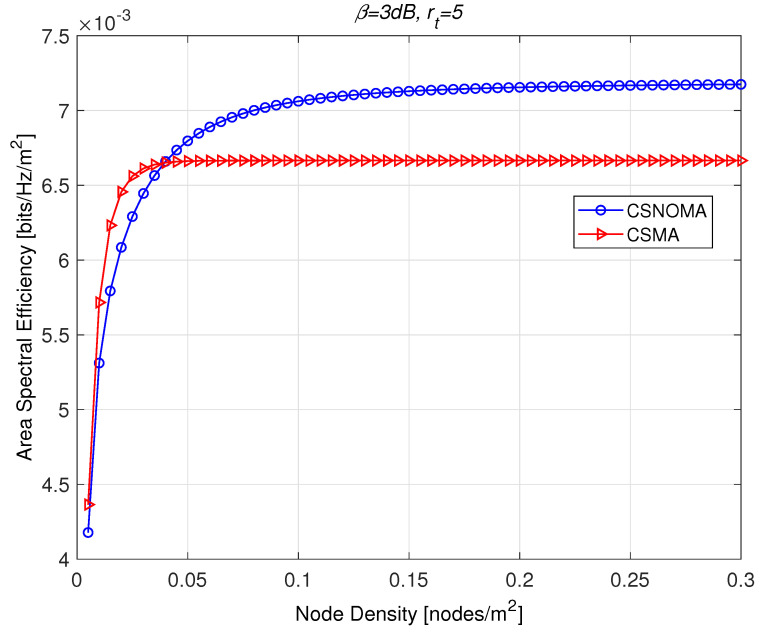
The area spectral efficiency of CSNOMA and carrier sense multiple access (CSMA) as a function of the node density. The target SIR β is 3 dB and the transmission distance $r_{t}$ is 5. As the network becomes dense, CSNOMA outperforms CSMA.

In the Figure 10, ASE of CSNOMA and CSMA is illustrated as a function of the node density. The target SIR β is 3 dB and the transmission distance $r_{t}$ is 5. As the network becomes dense, CSNOMA outperforms CSMA. Each value of ASE is evaluated with the optimal carrier sensing range that is computed for each of corresponding node density. In a sparse network, CSMA shows higher performance than CSNOMA. In this case, the smaller carrier sensing range would be applied to allow more transmitters can transmit simultaneously. This means that the network is not interference-limited and the cancelable distance $r_{c}$ becomes very small. Thus, CSNOMA did not work properly. On the other hand, as node density increases, CSNOMA outperforms CSMA. ASE of CSMA becomes saturated, while that of CSNOMA increases steadily. In the dense situation, the carrier sensing range would be widened, and the concurrent transmissions in the CSMA regime is highly restricted. In the CSNOMA regime, the cancelable strong interferers prevail, and IC are executed frequently. This leads to a performance improvement.

**Figure 11:**
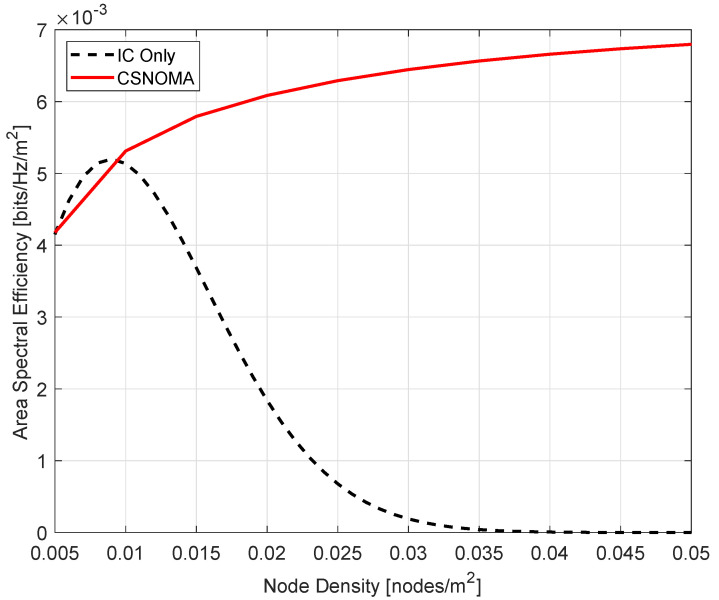
The area spectral efficiency of IC Only and CSNOMA as a function of the node density. The target SIR β is 3 dB and the transmission distance $r_{t}$ is 5.

We verify if CSNOMA improves IC performance by comparing the situation of IC Only (There is no MAC, only IC is executed.) and CSNOMA in the spatially randomly distributed network. We examine how ASE varies in each case while increasing the node density from 0.005 to 0.05. The target SIR β is 3 dB and the transmission distance $r_{t}$ is 5. As shown in Figure 11, when the node density is low, the performance of IC Only is slightly better. However, as the node density gradually increases, the performance of CSNOMA continues to increase, whereas IC Only’s performance decreases severely. This is because when the node density is low, the aggregate interference is small, so nodes on the network can communicate smoothly without MAC. In this case, as CSNOMA prevents simultaneous transmission of nodes due to carrier sensing, the initial performance is low. However, as the network becomes more dense, the number of nodes that want to communicate increases, and the aggregate interference becomes very large. For IC to succeed with a high probability, the aggregate interference must be small. Using IC Only, the aggregate interference cannot be controlled, causing IC to fail and severe performance degradation. On the other hand, in the case of using CSNOMA, even if the node density increases, the aggregate interference does not increase significantly because the number of nodes that can be transmitted at the same time is limited, and CSNOMA allows simultaneous access by nodes who can obtain SINR as high as possible, so the ASE performance improves. Furthermore, from the perspective of the transmitter, energy consumption can be reduced due to CSNOMA. The performance degradation of IC Only in a dense network is serious. A low ASE at very high node density means that almost all transmissions fail. If the transmission fails, the transmitter usually retransmits. Even if retransmission fails, retransmission is attempted several times. This causes great energy consumption. On the other hand, CSNOMA maintains relatively stable performance even when the network becomes denser. Transmitters often succeed in transmission, and because they do not retransmit, they can consume less energy.

To verify the performance of reactive mode and proactive mode, we conduct a computer simulation. The transmission distance is fixed as 5. The carrier sensing range varies from 5 to 10. Node density λ is 0.02. The target SIR threshold is 3 dB. To focus the effect of the spatial randomness, the simple path-loss channel model is adopted in the simulation.

**Figure 12:**
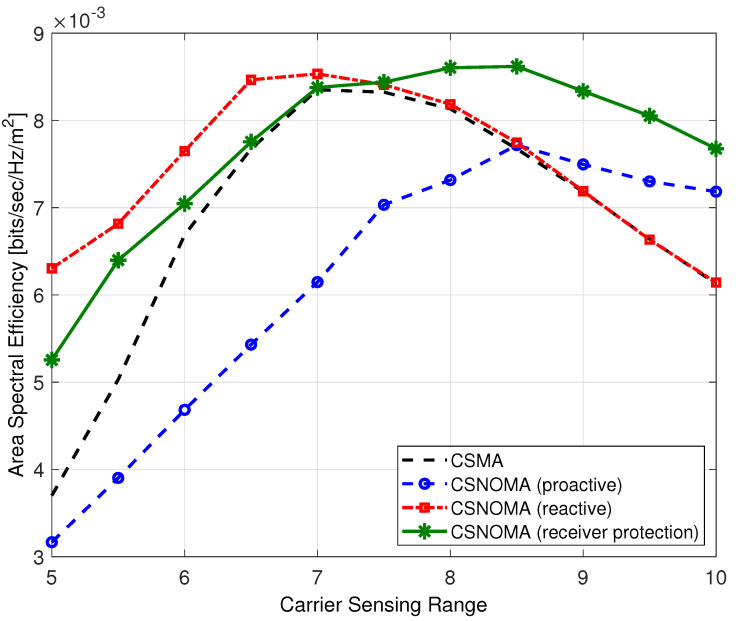
The area spectral efficiency of variants of CSNOMA and CSMA as a function of the carrier sensing range ($r_{t}$ = 5, β = 3 dB, λ = 0.02).

Figure 12 shows ASE of different modes of CSNOMA. With a small carrier sensing range, the hidden node problem frequently arises, and performance of reactive mode is superior. On the other hand, proactive mode shows poor performance with small carrier sensing range. As the carrier sensing range increases, exposed node problem occurs frequently, and the performance of proactive mode is improved. The receiver protection mode shows acceptable performance with various carrier sensing range.

**Figure 13:**
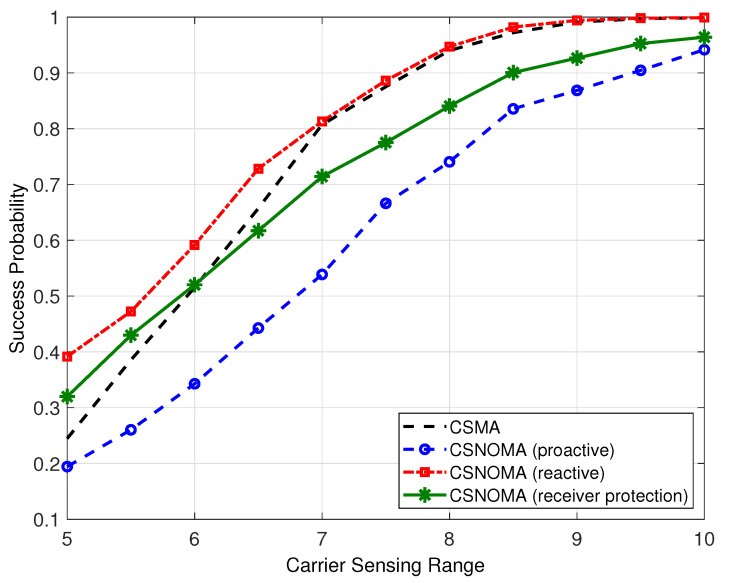
The success probability of variants of CSNOMA and CSMA as a function of the carrier sensing range ($r_{t}$ = 5, β = 3 dB, λ = 0.02).

Even though CSNOMA shows higher ASE performance, it may show poor success probability performance. Depending on applications, achieving high-level success probability is required. In that case, CSNOMA may be not preferred. Figure 13 shows the success probability of variants of CSNOMA and CSMA as a function of the carrier sensing range. The reactive mode shows superior performance than CSMA; the proactive and receiver protection modes do not. This is because the reactive mode utilizes the same amount of concurrent transmitting nodes. With small carrier sensing range, CSMA suffers the hidden node problem, while the reactive mode solves the problem and increases success probability. However, with wide carrier sensing range, hidden node problem rarely occurs, and the reactive mode operates like CSMA.

**Figure 14:**
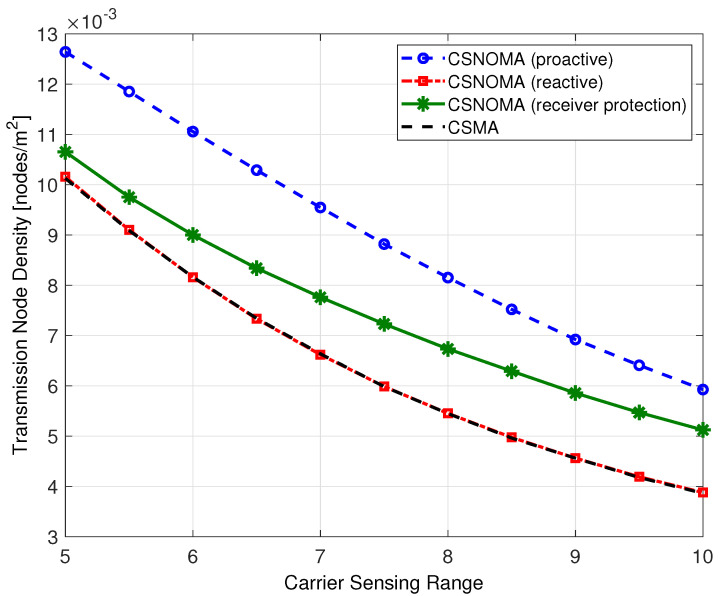
Transmission node density of variants of CSNOMA and CSMA as a function of the carrier sensing range ($r_{t}$ = 5, β = 3 dB, λ = 0.02).

Figure 14 shows the transmission node density as a function of the carrier sensing range. As shown in Figure 14, proactive and receiver protection modes utilize more transmitting nodes than reactive mode and CSMA. Therefore, the interference level increases and success probability decreases.

**Figure 15:**
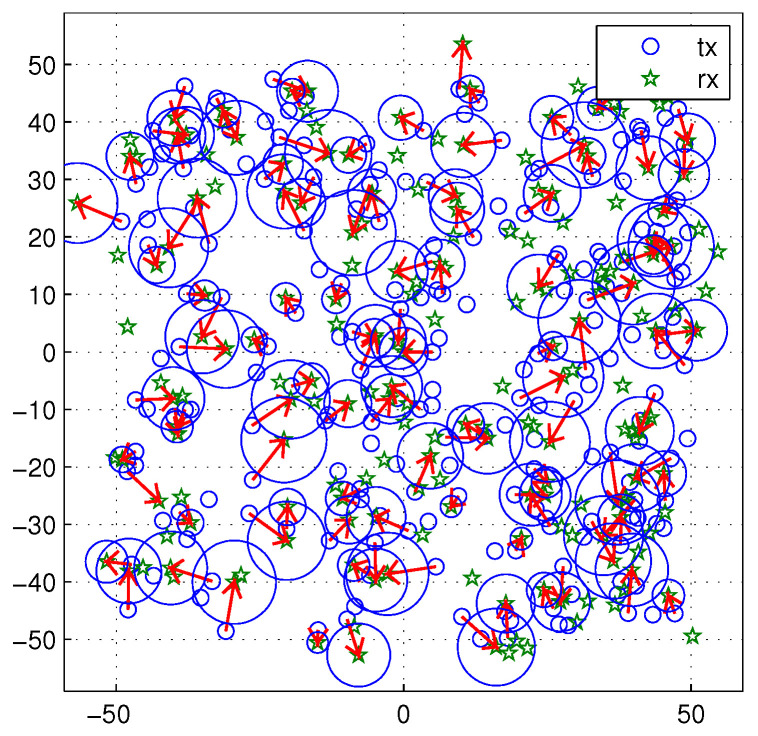
A snapshot of the network topology. The transmission distance varies from 2 to 9. The carrier sensing range is fixed as 5. Node density is 0.02. The cancelable distance $r_{c}$ varies (blue circles).

In real wireless networks, the carrier sensing range is usually fixed as a factory setting. Because the transmitters and receivers move in the network, it is hard to optimize the carrier sensing range all the time. To simulate this situation, the carrier sensing range is fixed as 5. The transmission distance varies from 2 to 9. Node density λ is 0.02. The variable communication distance also makes the cancelable distance variable. Figure 15 shows a snapshot of the network topology. In Figure 15, the variable size of circles represent the cancellable regions. The arrow line means the transmission be in progress.

**Figure 16:**
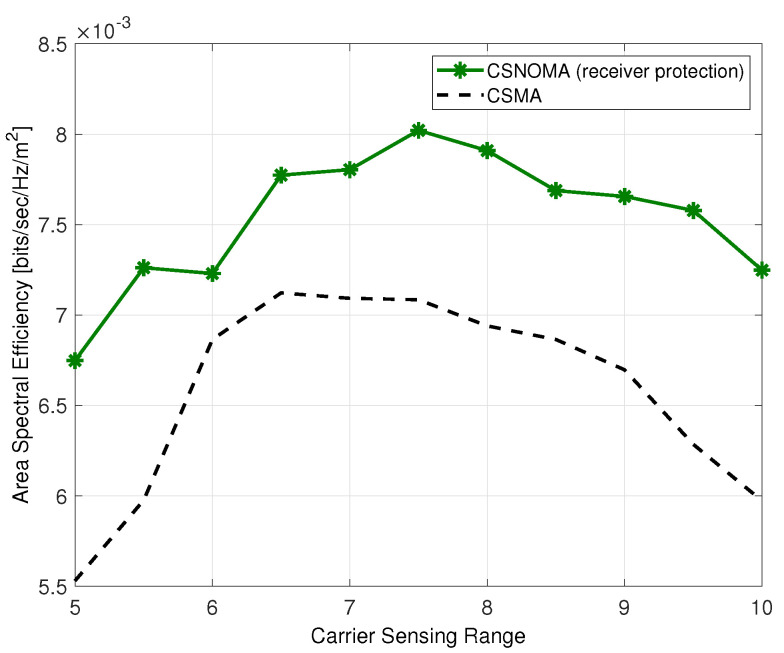
The area spectral efficiency of CSNOMA and CSMA as a function of the carrier sensing range (β = 3 dB, λ = 0.02). The transmission distance varies from 2 to 9.

Figure 16 shows ASE of CSNOMA and CSMA as a function of the carrier sensing range. As shown in Figure 16, the performance of CSNOMA is improved with receiver protection mechanism.

## 6. Concluding Remarks

In this paper, we investigated the possibility of cross-layer design of MAC with considering PHY and MAC layers. We proposed a new multiple access scheme, Carrier Sense Non-Orthogonal Multiple Access (CSNOMA). The proposed scheme utilized the interference cancellation technique, and shows better area spectral efficiency (ASE) performance than the CSMA scheme. The backward compatibility is important in new multiple access scheme design. An algorithm to derive an optimal carrier sensing range that maximizes ASE is presented through analysis according to the stochastic geometric theory. We designed the practical signaling process and the control packet frame structure compatible with IEEE 802.11 DCF mode in order to colocate the node applying CSNOMA and the node applying IEEE 802.11 DCF. In this protocol design, additional information is exchanged through hierarchical modulated CTS messages to reduce signaling overhead. Through this, higher ASE was achieved by finding nodes with good channel condition and performing IC. We also proposed a reactive mode that operates conservatively to reduce signaling overhead and energy consumption. The reactive mode was effective when a hidden node problem occurred as the carrier sensing range was relatively short. Although the transmission probability of a node that has seized a transmission opportunity through carrier sensing may be high, a node that is activated by receiving additional information from CTS is not protected by the carrier sensing range, so the transmission success rate may decrease. To compensate for this case, a receiver protection mode that conservatively performs node activation was presented. Our research direction is to build a testbed by implementing CSNOMA on real equipment. Through this, we will practically explore the convergence of the PHY layer and the MAC layer, and continue to try to combine with other PHY technologies.

## Figures and Tables

**Table 1:** Summary of related work.

Paper	Aim	Proposed Solution	Pros	Cons
[22]	Concurrent transmissions, multipacket reception (MPR) at the receiver to combat interference	Conflict set graph for the interference with successive interference cancellation (SIC)	Improve MPR	Difficult to characterize the link interface under SINR model
[23,24]	Concurrent transmissions, MPR at the receiver to combat interference	Scheduling scheme to improve bound	Improve the bound	Not efficient scheme to achieve good scheduling
[25]	Concurrent transmissions, MPR at the receiver to combat interference	Fair uplink scheduling algorithm	Increase network throughput	Low density network
[26]	Design SIC-aware MAC	Novel SIC-aware MAC protocol	Increase throughput by 1.5	Subjected to selfish behavior of users
[27]	Close the gap between throughput capacity in the physical model	Investigate network scaling law	Low power is attained to achieve higher throughput	Simplified the interference cancellation condition
[28]	Comparison on successive interference cancellation and Joint detection (JD)	Benefits of JD surpass those of SIC	JD provide significant outage benefit regardless of the SIR threshold	JD depend largely on good code schemes and more complicated hardware
[29]	Study the improvement of transmission capacity obtained with SIC	Closed-form upper and lower bounds for Transmission Capacity (TC) of ad hoc network with SIC receivers	It showed imperfections in the interference cancellation rapidly degrade its usefulness	The network density largely determines the efficacy of SIC
[30]	Summarize new analytical tools of TC (Survey paper)	Contributions developed metric for transmission capacity	Show improvement in transmission capacity	Limited information in MAC design issue
[31]	Examine interaction between the physical and medium access control layers	Cross-layer design between PHY and MAC	Quantify the effectiveness of signal processing on multipacket reception	Less practical
[32]	A MAC Protocol for Multi-Packet Ad-hoc Wireless Network Utilizing Multi-Antenna	A novel multi-antenna utilization scheme for avoiding the packet congestion	Improve throughput, decreases transmission delay in the relay node	Only works with multiple antennas
[33]	MAC for MPR	Markovian analysis was used to evaluate performance	Ability to capture few packets simultaneously	Can lead to unfairness when nodes are spatially distributed
[34]	Enable the coexistence utilize the MPR capability to maximize throughput	New MPR MAC protocol	Improves throughput	Spatially random topology is not considered
[35]	To construct a femtocell cluster sharing the same frequency band	Interference management scheme	Reduce cross-tier interference in the downlink	The performance of FUEs decreased
[36]	Analysis of IC scheme in a DS/CDMA system	Analyze a simple successive interference cancellation scheme for coherent BPSK modulation	Extend the analysis for a noncoherent modulation scheme	Limited consideration on analyzing the processing delay involved and the practical implementation of the interference canceler
[37]	Maximize the overall capacity to estimate error	Power control strategy	Power control is a key element in allowing a SIC system to achieve high performance in practice	Requires highly accurate estimates for the amplitude and phase of each user’s signal
[38]	High packet loss rate and poor spatial reuse	Algorithm for interference cancellation	Reduced packet loss and increase spatial reuse	Not fully combined IC into MAC
[39]	Performance study of CSMA	CSMA for single packet reception is designed	SIC provides many new transmission opportunities	Not suitable to combining IC capability

**Table 2:** Key mathematical notations.

Notation	Description
α	path loss exponent, α>2
β	target SIR threshold β>1
$r_{t}$	communication distance
$r_{s}$	carrier sensing distance
$r_{a}$	strong interferer distance
$r_{c}$	maximum cancellable distance
$b\left(o,r\right)$	a ball of radius *r* centered at origin
$a\left(o,r_{1},r_{2}\right)$	an annulus between $r_{1}$ and $r_{2}$
$I_{r_{1}}^{r_{2}}$	interference at typical receiver from $a\left(o,r_{1},r_{2}\right)$, $I_{r_{1}}=I_{r_{1}}^{\infty }$
A(x)	area of region *x*
N(x)	number of points in region *x*
λ	node density
$\lambda_{t}$	transmitting node density
$\lambda_{b}$	additional transmitting node density
Φ	a Poisson point process of intensity λ
$\left\{X_{i}\right\}$	a set of points in Φ
$\left|X_{i}\right|$	euclidian distance between $X_{i}$ and origin
$p_{s}$	transmission success probability
$p_{s}^{\mathrm{IC}\;-\;k}$	transmission success probability with *k*-times interference cancellation

**Table 3:** Classification of allowed/restricted transmission areas according to the distance from the receiver.

Distance	Type of Region
*r* > $r_{s}$	Transmission allowed region (a)
$r_{c}$ < *r* < $r_{s}$	Transmission restricted region (b)
*r* < $r_{c}$	Transmission allowed region (c)

## Appendix A

Let $G_{t}$ denote the channel gain of a typical link. Let $I_{r_{s}}$ denote the interference from the outside of the sensing range $r_{s}$, then $I_{r_{s}}=\sum_{X_{i}\in \Phi_{r_{s}}^{\infty }}G_{i}{\left|X_{i}\right|}^{-\alpha }P$, where $G_{i}$ denotes the channel gain of each interfering link, $\Phi_{r_{s}}^{\infty }$ is a set of interfering nodes outside of the sensing range $r_{s}$, $X_{i}$ is a point in $\Phi_{r_{s}}^{\infty }$, and $\left|X_{i}\right|$ is an euclidean distance between $X_{i}$ and origin. The success probability $p_{s}^{\text{non-IC}}$ can be expressed as follows,

(A1) $$\begin{array}{c} p_{s}^{\text{non-IC}}=\mathrm{Pr}\left[\frac{G_{t}r_{t}^{-\alpha }P}{I_{r_{s}}}\geq \beta \right]=\mathrm{Pr}\left[G_{t}\geq \frac{\beta r_{t}^{\alpha }}{P}I_{r_{s}}\right]=E_{I_{r_{s}}}\left[\exp\left(-\frac{\mu \beta r_{t}^{\alpha }}{P}I_{r_{s}}\right)\right]. \end{array}$$

Let $s=\frac{\mu \beta r_{t}^{\alpha }}{P}$, then $E_{I_{r_{s}}}\left[\exp\left(-sI_{r_{s}}\right)\right]$ is expressed as follows,

(A2) $$\begin{array}{cc} E_{I_{r_{s}}}\left[\exp\left(-sI_{r_{s}}\right)\right] & =\exp\left(-2\pi \left(\lambda_{t}+\lambda_{a}\right)\int_{r_{s}}^{\infty }\left(1-E_{G}\left[e^{-sGPv^{-\alpha }}\right]\right)vdv\right)\\ \; & =\exp\left(-2\pi \left(\lambda_{t}+\lambda_{a}\right)\int_{r_{s}}^{\infty }\left(\frac{\beta }{\beta +\frac{v^{\alpha }}{r_{t}^{\alpha }}}\right)vdv\right)\\ ∴p_{s}^{\text{non-IC}} & =\exp\left(-\pi \left(\lambda_{t}+\lambda_{a}\right)\sqrt{\beta }r_{t}^{2}\arctan\left(\frac{\sqrt{\beta }r_{t}^{2}}{r_{s}^{2}}\right)\right). \end{array}$$

## Appendix B

Let $G_{a}$ denote the channel gain of an additional link and let $r_{a}$ denote the distance between the additional transmitting node and the typical receiver. Then, the success probability allowing an additional transmission at the distance $r_{a}$ and executing SIC can be expressed as follows,

(A3) $$\begin{array}{cc} \; & E_{I_{r_{s}}}\left[\mathrm{Pr}\left[\frac{G_{t}r_{t}^{-\alpha }P}{I_{r_{s}}}\geq \beta ,\frac{G_{a}r_{a}^{-\alpha }P}{I_{r_{s}}+G_{t}r_{t}^{-\alpha }P}\geq \beta \right]\right]\\ \; & \geq E_{I_{r_{s}}}\left[\mathrm{Pr}\left[G_{t}\geq \frac{\beta r_{t}^{\alpha }I_{r_{s}}}{P}\right]\right]E_{I_{r_{s}}}\left[E_{G_{t}}\left[\mathrm{Pr}\left[G_{a}\geq \frac{\beta r_{a}^{\alpha }I_{r_{s}}}{P}+\frac{\beta G_{t}r_{a}^{\alpha }}{r_{t}^{\alpha }}\right]\right]\right]\\ \; & =E_{I_{r_{s}}}\left[\exp\left(-\frac{\mu \beta r_{t}^{\alpha }}{P}I_{r_{s}}\right)\right]E_{I_{r_{s}}}\left[\exp\left(-\frac{\mu \beta r_{a}^{\alpha }}{P}I_{r_{s}}\right)\right]E_{G_{t}}\left[\exp\left(-\frac{\mu \beta G_{t}r_{a}^{\alpha }}{r_{t}^{\alpha }}\right)\right]\\ \; & =E_{I_{r_{s}}}\left[\exp\left(-\frac{\mu \beta r_{t}^{\alpha }}{P}I_{r_{s}}\right)\right]E_{I_{r_{s}}}\left[\exp\left(-\frac{\mu \beta r_{a}^{\alpha }}{P}I_{r_{s}}\right)\right]\frac{r_{t}^{\alpha }}{\beta r_{a}^{\alpha }+r_{t}^{\alpha }}. \end{array}$$

Let us assume that the distance $r_{a}$ is a nearest neighbor distance. Then, the lower bound of the success probability $p_{s}^{\mathrm{IC}}$ is

(A4) $$\begin{array}{cc} p_{s}^{\mathrm{IC}} & \geq \int_{0}^{\beta^{-\frac{1}{\alpha }}r_{t}}E_{I_{r_{s}}}\left[\exp\left(-\frac{\mu \beta r_{t}^{\alpha }}{P}I_{r_{s}}\right)\right]E_{I_{r_{s}}}\left[\exp\left(-\frac{\mu \beta r_{a}^{\alpha }}{P}I_{r_{s}}\right)\right]\\ \; & \;\times \frac{r_{t}^{\alpha }}{\beta r_{a}^{\alpha }+r_{t}^{\alpha }}2\left(\lambda -\lambda_{t}\right)\pi r_{a}\exp\left(-\left(\lambda -\lambda_{t}\right)\pi r_{a}^{2}\right)dr_{a}. \end{array}$$

Let $s_{t}=\frac{\mu \beta r_{t}^{\alpha }}{P}$ and $s_{c}=\frac{\mu \beta r_{c}^{\alpha }}{P}$,

(A5) $$\begin{array}{cc} p_{s}^{\mathrm{IC}} & \geq \int_{0}^{\beta^{-\frac{1}{\alpha }}r_{t}}E\left[\exp\left(-s_{t}I_{r_{s}}\right)\right]E\left[\exp\left(-s_{a}I_{r_{s}}\right)\right]\\ \; & \;\times \frac{2\left(\lambda -\lambda_{t}\right)\pi r_{a}r_{t}^{\alpha }}{\beta r_{a}^{\alpha }+r_{t}^{\alpha }}\exp\left(-\left(\lambda -\lambda_{t}\right)\pi r_{a}^{2}\right)dr_{a}. \end{array}$$

The value $E\left[\exp\left(-s_{t}I_{r_{s}}\right)\right]$ is

(A6) $$\begin{array}{cc} E\left[\exp\left(-s_{t}I_{r_{s}}\right)\right] & =\exp\left(-2\pi \left(\lambda_{t}+\lambda_{a}\right)\int_{r_{s}}^{\infty }\left(1-E_{G}\left[e^{-s_{t}GPv^{-\alpha }}\right]\right)vdv\right)\\ \; & =\exp\left(-2\pi \left(\lambda_{t}+\lambda_{a}\right)\int_{r_{s}}^{\infty }\frac{\beta }{\beta +{\left(v/r_{t}\right)}^{\alpha }}vdv\right). \end{array}$$

Let $u={\left(\frac{v}{r_{t}\beta^{1/\alpha }}\right)}^{2}$, then,

(A7) $$\begin{array}{cc} E\left[\exp\left(-s_{t}I_{r_{s}}\right)\right] & =\exp\left(-2\pi \left(\lambda_{t}+\lambda_{a}\right)\int_{{\left(\frac{r_{s}}{r_{t}}\right)}^{2}\frac{1}{\beta^{2/\alpha }}}^{\infty }\frac{\beta }{\beta +\beta u^{\alpha /2}}\frac{r_{t}^{2}\beta^{2/\alpha }}{2}du\right)\\ \; & =\exp\left(-\pi \left(\lambda_{t}+\lambda_{a}\right)r_{t}^{2}\beta^{2/\alpha }\int_{{\left(\frac{r_{s}}{r_{t}}\right)}^{2}\frac{1}{\beta^{2/\alpha }}}^{\infty }\frac{1}{1+u^{\alpha /2}}du\right)\\ \; & =\exp\left(-\pi \left(\lambda_{t}+\lambda_{a}\right)\sqrt{\beta }r_{t}^{2}\arctan\left(\frac{\sqrt{\beta }r_{t}^{2}}{r_{s}^{2}}\right)\right). \end{array}$$

The value $E\left[\exp\left(-s_{a}I_{r_{s}}\right)\right]$ can be found in a similar way. Then,

(A8) $$\begin{array}{c} E\left[\exp\left(-s_{a}I_{r_{s}}\right)\right]=\exp\left(-\pi \left(\lambda_{t}+\lambda_{a}\right)\sqrt{\beta }r_{a}^{2}\arctan\left(\frac{\sqrt{\beta }r_{a}^{2}}{r_{s}^{2}}\right)\right). \end{array}$$

Finally, we get the result.

## References

[1] Cisco (2020). Cisco Annual Internet Report (2018–2023) White Paper.

[2] Yu H., Afzal M.K., Zikria Y.B., Rachedi A., Fitzek F.H. (2020). Tactile Internet: Technologies, test platforms, trials, and applications. Future Gener. Comput. Syst..

[3] Alouini M.S., Goldsmith A.J. (1999). Area spectral efficiency of cellular mobile radio systems. IEEE Trans. Veh. Technol..

[4] Baccelli F., Błaszczyszyn B., Mühlethaler P. (2006). An Aloha protocol for multihop mobile wireless networks. IEEE Trans. Inf. Theory.

[5] Kim D.M., Kim S.L. (2013). An iterative algorithm for optimal carrier sensing threshold in random CSMA/CA wireless networks. IEEE Commun. Lett..

[6] Bianchi G. (2000). Performance analysis of the IEEE 802.11 distributed coordination function. IEEE J. Sel. Areas Commun..

[7] Saito Y., Kishiyama Y., Benjebbour A., Nakamura T., Li A., Higuchi K. Non-orthogonal multiple access (NOMA) for cellular future radio access. Proceedings of the IEEE Vehicular Technology Conference (VTC Spring).

[8] 3GPP (2018). Study on Non-Orthogonal Multiple Access (NOMA) for NR. TR 38.812. https://portal.3gpp.org/desktopmodules/Specifications/SpecificationDetails.aspx?specificationId=3236.

[9] Vaezi M., Schober R., Ding Z., Poor H.V. (2019). Non-orthogonal multiple access: Common myths and critical questions. IEEE Wirel. Commun..

[10] Del-Valle-Soto C., Mex-Perera C., Orozco-Lugo A., Lara M., Galván-Tejada G.M., Olmedo O. (2014). On the MAC/Network/Energy performance evaluation of wireless sensor networks: Contrasting MPH, AODV, DSR and ZTR routing protocols. Sensors.

[11] Yan J., Zhou M., Ding Z. (2016). Recent advances in energy-efficient routing protocols for wireless sensor networks: A review. IEEE Access.

[12] Andrews J.G. (2005). Interference cancellation for cellular systems: A contemporary overview. IEEE Wirel. Commun..

[13] Jafar S.A. (2011). Interference Alignment: A New Look at Signal Dimensions in a Communication Network.

[14] Costa M. (1983). Writing on dirty paper. IEEE Trans. Inf. Theory.

[15] Katti S., Gollakota S., Katabi D. (2007). Embracing wireless interference: Analog network coding. ACM SIGCOMM Comput. Commun. Rev..

[16] Popovski P., Yomo H. Physical network coding in two-way wireless relay channels. Proceedings of the 2007 IEEE International Conference on Communications.

[17] Jain M., Choi J.I., Kim T., Bharadia D., Seth S., Srinivasan K., Levis P., Katti S., Sinha P. Practical, real-time, full duplex wireless. Proceedings of the 17th Annual International Conference on Mobile Computing and Networking.

[18] Bharadia D., McMilin E., Katti S. Full duplex radios. Proceedings of the ACM SIGCOMM 2013 Conference on SIGCOMM.

[19] Hong S., Brand J., Choi J.I., Jain M., Mehlman J., Katti S., Levis P. (2014). Applications of self-interference cancellation in 5G and beyond. IEEE Commun. Mag..

[20] Jiang Z., Zhou M. (2007). Spread spectrum MAC protocol with dynamic rate and collision avoidance for mobile ad hoc network. IEEE Trans. Veh. Technol..

[21] Zheng M., Lin J., Liang W., Yu H. (2015). A priority-aware frequency domain polling MAC protocol for OFDMA-based networks in cyber-physical systems. IEEE/CAA J. Autom. Sin..

[22] Lv S., Wang X., Zhou X. Scheduling under SINR Model in Ad Hoc Networks with successive interference cancellation. Proceedings of the 2010 IEEE Global Telecommunications Conference GLOBECOM 2010.

[23] Lv S., Zhuang W., Wang X., Zhou X. Scheduling in wireless ad hoc networks with successive interference cancellation. Proceedings of the IEEE INFOCOM 2011.

[24] Lv S., Zhuang W., Wang X., Zhou X. (2011). Link scheduling in wireless networks with successive interference cancellation. Comput. Netw..

[25] Mollanoori M., Ghaderi M. Fair and efficient scheduling in wireless networks with successive interference cancellation. Proceedings of the 2011 IEEE Wireless Communications and Networking Conference.

[26] Mollanoori M., Ghaderi M. On the performance of successive interference cancellation in random access networks. Proceedings of the 2012 9th Annual IEEE Communications Society Conference on Sensor, Mesh and Ad Hoc Communications and Networks (SECON).

[27] Sadjadpour H.R., Wang Z., Garcia-Luna-Aceves J.J. (2010). The capacity of wireless ad hoc networks with multipacket reception. IEEE Trans. Commun..

[28] Blomer J., Jindal N. Transmission capacity of wireless ad hoc networks: Successive interference cancellation vs. joint detection. Proceedings of the IEEE International Conference on Communications (ICC).

[29] Weber S.P., Andrews J.G., Yang X., de Veciana G. (2007). Transmission capacity of wireless ad hoc networks with successive interference cancellation. IEEE Trans. Inf. Theory.

[30] Weber S., Andrews J.G., Jindal N. (2010). An overview of the transmission capacity of Wireless Networks. IEEE Trans. Commun..

[31] Tong L., Zhao Q., Mergen G. (2001). Multipacket reception in random access wireless networks: From signal processing to optimal medium access control. IEEE Commun. Mag..

[32] Sato N., Fujii T. A MAC protocol for multi-packet ad-hoc wireless network utilizing multi-antenna. Proceedings of the 6th IEEE Consumer Communications and Networking Conference (CCNC).

[33] Celik G.D., Zussman G., Khan W.F., Modiano E. (2010). MAC for networks with multipacket reception capability and spatially distributed nodes. IEEE Trans. Mob. Comput..

[34] Lee H., Lim C.C., Choi J. (2011). A refined MAC protocol with multipacket reception for wireless networks. Wirel. Commun. Mob. Comput..

[35] Ghosh A., Mangalvedhe N., Ratasuk R., Mondal B., Cudak M., Visotsky E., Thomas T.A., Andrews J.G., Xia P., Jo H.S. (2012). Heterogeneous cellular networks: From theory to practice. IEEE Commun. Mag..

[36] Patel P., Holtzman J. (1994). Analysis of a simple successive interference cancellation scheme in a DS/CDMA system. IEEE J. Sel. Areas Commun..

[37] Andrews J.G., Meng T.H. (2003). Optimum power control for successive interference cancellation with imperfect channel estimation. IEEE Trans. Wirel. Commun..

[38] Halperin D., Anderson T., Wetherall D. Taking the sting out of carrier sense: Interference cancellation for wireless LANs. Proceedings of the 14th ACM International Conference on Mobile Computing and Networking (MOBICOM ’08).

[39] Lv S., Zhuang W., Wang X., Liu C., Hu X., Sun Y., Zhou X. A performance study of CSMA in wireless networks with successive interference cancellation. Proceedings of the 2012 IEEE International Conference on Communications (ICC).

[40] Baccelli F., Błaszczyszyn B. (2009). Stochastic Geometry and Wireless Networks, Volume I: Theory.

[41] Haenggi M., Ganti R.K. (2009). Interference in large wireless networks. Found. Trends Netw..

[42] Jiang H., Wilford P.A. (2005). A hierarchical modulation for upgrading digital broadcast systems. IEEE Trans. Broadcast..

[43] Park J.M., Kim S.L., Choi J. (2010). Hierarchically modulated network coding for asymmetric two-way relay systems. IEEE Trans. Veh. Technol..

[44] Nguyen H.Q., Baccelli F., Kofman D. A stochastic geometry analysis of dense IEEE 802.11 networks. Proceedings of the IEEE INFOCOM 2007—26th IEEE International Conference on Computer Communications.

[45] Stoyan D., Kendall W., Mecke J. (1995). Stochastic Geometry and Its Applications.

[46] Yuan Y.X. (2008). Step-sizes for the gradient method. AMS IP Stud. Adv. Math..

